# Access to Healthcare for Immigrant Children in Canada

**DOI:** 10.3390/ijerph17093320

**Published:** 2020-05-10

**Authors:** Bukola Salami, Alleson Mason, Jordana Salma, Sophie Yohani, Maryam Amin, Philomena Okeke-Ihejirika, Tehseen Ladha

**Affiliations:** 1 Faculty of Nursing, University of Alberta, Edmonton, AB T6G 1C9, Canada; mason@ualberta.ca (A.M.); sjordana@ualberta.ca (J.S.); 2 Faculty of Education, University of Alberta, Edmonton, AB T6G 2G5, Canada; sophie.yohani@ualberta.ca; 3 Faculty of Medicine and Dentistry, University of Alberta, Edmonton, AB T6G 2R7, Canada; sharifzd@ualberta.ca (M.A.); tehseen.ladha@ualberta.ca (T.L.); 4 Faculty of Arts, University of Alberta, Edmonton, AB T6G 2R7, Canada; pokeke@ualberta.ca

**Keywords:** access to healthcare, Alberta, Canada, child health, immigrant health, immigration, migrant health, migration

## Abstract

Immigrants experience poorer health outcomes than nonimmigrants in Canada for several reasons. A central contributing factor to poor health outcomes for immigrants is access to healthcare. Previous research on access to healthcare for immigrants has largely focused on the experience of immigrant adults. The purpose of this study was to investigate how immigrants access health services for their children in Alberta, Canada. Our study involved a descriptive qualitative design. Upon receiving ethics approval from the University of Alberta Research Ethics Board, we invited immigrant parents to participate in this study. We interviewed 50 immigrant parents, including 17 fathers and 33 mothers. Interviews were audio recorded, transcribed, and analyzed according to the themes that emerged. Findings reveal that systemic barriers contributed to challenges in accessing healthcare for immigrant children. Participants identified several of these barriers—namely, system barriers, language and cultural barriers, relationship with health professionals, and financial barriers. These barriers can be addressed by policymakers and service providers by strengthening the diversity of the workforce, addressing income as a social determinant of health, and improving access to language interpretation services.

## 1. Introduction

International migration is an increasing global phenomenon. The United Nations estimated that in 2015 there were almost 244 million international migrants, representing 3.3% of the world’s population [1]. Drivers of migration include a quest for a better economic outcome as well as political instability [1]. Unfortunately, immigrants often experience several challenges to their health and wellbeing upon arrival in host societies [2], including in Canada. Canada has made successive changes to its immigration policy that have resulted in increased annual immigrant intake, which underscores the need to address immigrants’ health concerns. For instance, Canada’s refugee population has taken new shape with the settlement of over 40,000 Syrian refugees between November 2015 and January 2017 [3]. In 2016, 37.5% of the total population of youth under 15 years old had at least one parent who was born outside of Canada [4].

Disparities exist in the health status of immigrant children. Postmigration experience, and immigration policies can have a significant impact on healthcare access. Documented evidence in diverse countries across the globe indicates that immigrants, including children, generally arrive in many countries healthier than the host population. The criteria for immigrant selection, including Canada’s, favor healthy candidates [5]. Diverse social determinants of health, including income, employment, and community belonging, have been implicated in the decline of immigrants’ health [6]. Barriers to accessing healthcare have also been implicated as a contributing factor to this decline [5].

This paper focuses on access to healthcare for immigrants in Alberta, Canada. Permanent residents of Canada, Canadian citizens, international students, and temporary foreign workers who will reside in Alberta for at least 12 consecutive months can apply for and receive the Alberta Health Care Insurance Plan (AHCIP) for themselves and their children [7]. The AHCIP covers physician services, laboratory and diagnostic tests as well as a few select dental, optometry and podiatry services. It does not cover routine dental procedures, prescription drugs, nor services provided by clinical psychologists [8]. Refugees are given health coverage under the Interim Federal Health Program (IFHP) until they become eligible for provincial or territorial health insurance coverage. This gives them access to physician services, laboratory, and diagnostic tests, limited dental and vision care, and some prescription drugs [9]. Low income families can apply for welfare benefits such as income support and Alberta Child Health Benefit which covers routine dental and eyecare for children as well as prescription drugs [10].

Cultural differences, lack of social support, socioeconomic status, health system structure, lack of universal healthcare coverage upon initial arrival in Canada, language barriers, limited knowledge of health services, treatment preferences, and geographic distance to health services are all potential barriers to accessing healthcare services for immigrants in Canada [11,12,13,14,15]. Research demonstrates that language is the dominant barrier as many immigrants find it difficult to understand healthcare providers when services are not available in their first language and often do not know if interpretation is available to them [16,17,18,19,20,21]. Bowen [22] noted that few Canadian health regions and institutions have policies making some type of interpretation service available to every patient who needs it. Language barriers lead to the misdiagnosing of health challenges by physicians, patients being treated for the wrong illnesses, and cause immigrants and refugees to mistrust their physicians, ultimately straining the relationship between patients and their healthcare providers [23,24]. Additionally, even where patients have access to translators they are sometimes unavailable when patients have appointments or are deemed inappropriate by patients depending on the health concern being discussed, for instance, some female patients are reluctant to discuss issues pertaining to their reproductive system using male interpreters [25]. Another key barrier is the cost of services that are not covered by the government; some families do not have extended health insurance or other financial resources to pay for medical equipment or dental care for their children [17,26,27,28,29,30]. Many families also lack information about the range of health services and funding support for health available to them [17,21]. Furthermore, migration status has been associated with the likelihood of receiving poor care. Immigrant women in Montreal and Toronto were twice as likely as Canadian-born women to not have their and their infants’ postpartum health concerns addressed [31]. These concerns included postpartum depression, visualizing self-harm, mothers feeling back pain, children having vitamin D deficiency, and not having a change of clothes and bedding for babies [31]. Likewise, Gannon et al. [32] in studying the impact of maternal country of birth on post partum health care access compared access by Canadian- born women and immigrants. They found that immigrant women were less satisfied with health services than the Canadian- born and were less likely have their emotional and physical healthcare needs met. Other studies such as Lasser et al. [33] also found that, in Canada and the United States of America, immigrant patients were more likely to be dissatisfied with the quality of healthcare they received than the natives.

Government resettlement programs tend to place refugees in suburban areas or in areas where they do not have access to public transportation and this makes it difficult to commute to health services which are typically in urban centers [25] Many refugees do not have the financial resources to purchase a car, especially if they have recently arrived in Canada, thus the combination of where they have been placed to live and their low income makes transportation a barrier to medical services. Many Canadian-born families in suburban areas would have access to or own at least one car which they can use to travel to urban centers. Moreover in their study of immigrants’ healthcare access in Ontario, Goel et al. [34] reported that the mandatory three-month period to access free health services under the provincial health insurance caused new immigrants to delay seeking healthcare for themselves and their sick children for fear of not being able to pay out of pocket for services. This contributed to mental distress and could lead to negative health outcomes for some patients depending on their ailment. Like Ontario, Alberta also has a three-month waiting period before new immigrants can access provincial health insurance and it is highly probable that immigrants in that province experience similar challenges. These barriers contribute to a decreased use of health services by immigrants [35].

Notably, most studies on barriers to access to healthcare in Canada focus on immigrant adults. We found limited studies on access to healthcare for immigrant children in western Canada [16,17,29,30,36]. While we found limited studies on access to primary healthcare, health promotion, and mental health services for immigrant and refugee children, research on oral health of immigrant children in western Canada showed barriers to access to dental healthcare for immigrant and refugee children in Alberta [16,37]. We suspect that the results on access to healthcare for children versus adults may be different as children often use pediatric services rather than adult services which may not be available in all communities. Also, children’s services often involve the integration of family (e.g., parents and caregivers) into care. The purpose of this study was to investigate how immigrants, of various immigrant categories, access health services for their children in Alberta. We seek to answer the following research questions:

1. What are the experiences of immigrant parents in accessing health services for their children in Alberta?
2. What are the barriers to access to healthcare for immigrant children in Alberta?

## 2. Materials and Methods

We used a qualitative descriptive design for this study. Qualitative description draws on the tenets of naturalistic inquiry and provides a comprehensive summary of a phenomenon in the everyday context of that phenomenon [38,39]. Thus, qualitative description produces findings closer to the data than other qualitative methodological approaches such as phenomenology and grounded theory. Qualitative description also includes analysis and interpretation of data [39].

Upon receiving ethics approval from the University of Alberta Research Ethics Board, we invited immigrant parents to participate in this study. Fifty participants were recruited through immigrant-serving agencies and clinics that are regularly accessed by immigrant populations, as well as snowball sampling. We invited participants to engage in a one-hour interview at a time and place that was convenient to them. Participants were given time to review the informed consent form, ask questions, and sign the form. Interviews were conducted by two research assistants who had training in qualitative research and research ethics. One of these research assistants, an Afro-Caribbean immigrant, conducted most of the interviews. Before each interview, participants read an informed consent form, which included information on the study, confidentiality, right to withdraw, benefits, risks, compensation, etc. Any questions participants had were answered. Interviews lasted one hour and were conducted in a language of the participant’s choice. All but 14 interviews were completed in English. We used trained interpreters for the 14 interviews in other languages. Due to limited finances, we transcribed the English interpretation of these interviews.

All interviews were transcribed verbatim and data analysis was completed. Two members of the research team read five transcripts to familiarize themselves with the data. One of these two members was an associate professor with expertise in immigrant health, and the other was an immigrant graduate student. A coding tree was inductively created by identifying interesting features of the data with the aid of NVivo 12 software (QSR International, Burlington, MA, USA). The coding tree included the main codes and sub codes based on the initial five interviews. The two research team members compared notes and agreed on the main coding categories and sub codes. Then, the research assistant completed inductive coding of the data under the supervision of the senior research team member. The coding tree consisted of initial descriptive codes. Next, commonalities across the descriptive codes were identified, similar descriptive codes were clustered together and given a name to form themes and reviewed. We defined and named themes, and examined consistency and diversity within the data set. The final stage of the analysis was the synthesis and writing up of the results.

To ensure quality and increase the credibility of the data, we engaged in peer debriefing. Thick descriptions have been provided in these results in the form of verbatim quotes of participants’ descriptions of their experiences. We also kept an audit trail of our decisions throughout the research process.

## 3. Results

### 3.1. The Characteristics of Participants

In total, 50 immigrant parents participated in this study, 17 fathers and 33 mothers. Forty-three of these participants were married, three were separated, one was divorced, and three did not answer the question on marital status. Forty-six participants had a family doctor or pediatrician for their child, while four participants did not have one but largely relied on walk-in clinics. Participants were from diverse countries and migrated through diverse immigration routes. The average age of participants’ children was six years old. Further demographic data is available in Table 1. Below we present the main themes from our findings including: systemic barriers, language and cultural barriers, relationship to health professionals, and financial barriers. These themes represent factors that contribute to access to healthcare for immigrant children.

### 3.2. System Barriers

The most frequently identified barrier was the long wait times between making appointments and seeing health professionals, and the long wait times at health facilities. Parents mentioned that it took between two days to three months to see a pediatrician, which forced them to seek care from their family doctors, doctors at walk-in clinics, or emergency rooms when their children were ill. Additionally, when they sought assistance at the clinics and emergency rooms, they waited for many hours before seeing a doctor. Participants indicated that they waited between 45 minutes up to six hours to see a doctor at the emergency room.

The long wait times at emergency departments were a deterrent for some parents. Long wait times meant that parents had to take significant time away from work and other activities. Additionally, the sick child’s siblings missed school because parents cannot predict whether they will be finished at the doctor’s office in time to retrieve their other children from school.

For participants who worked or studied, opening times were sometimes inconvenient. Some family doctors/pediatricians work selected days during the week and are open only during regular office hours. Some participants found that these times posed a challenge because they needed to be at work or at school. They indicated that if doctors could operate outside regular business hours at some point during the week, it would allow them to access services without taking time off work. Further, inflexible opening hours may force parents to disrupt their children’s schooling to take them for medical appointments. Participants indicated that more flexible hours could allow them to better fit their children’s care into their schedule, which will ensure they get the care they need in a timely manner with minimal stress on them.

### 3.3. Language and Cultural Barriers

Language and lack of familiarity with the healthcare system were other prevalent barriers to accessing healthcare. Participants who were not fluent in English had difficulty having a conversation with medical professionals, which limited the extent to which they were able to communicate their children’s symptoms. It also limited their ability to understand, retain, and implement health professionals’ instructions. Through the help of an interpreter, one Chinese participant explained,

Okay, so that experience was really something for the family, because he was at work and his wife took the girl to emergency, but when she went to emergency, you know, she cannot speak the language, and the worker was not able to understand what’s going on with the daughter.

(Participant 037, Dad)

In this situation, seeking care for this child caused psychological strain because the parent did not have access to translation services at the healthcare provider. Some participants tried to address this problem by using family members and friends as interpreters. However, this strategy came with challenges of its own, including issues of confidentiality and trust as some people feared that the interpreters would divulge sensitive information to other community members. Regarding the use of interpreters Participant 030, a mother, stated,

So it’s an always and 100 percent first barrier they face. That is, the first thing which hold them back, and then they started calling friends here and there. And though interpretation and translators are available, but they don’t trust. There is no trust in the translators and interpreters nowadays, because they think because always translators and interpreters that is from their community and they don’t trust them. They think maybe they are giving us wrong information. I don’t know, there is an issue of trust always with the interpretation.

Even in instances where trust was not an issue, untrained interpreters may not translate information between healthcare providers and parents accurately as they may not know how to translate some medical terms or how to convey cultural nuances or differences. Medical translators are specially trained professionals and possess knowledge and skills that a layperson does not have. The absence of professional medical translators can constrain the ability of the healthcare professional to accurately diagnose and treat the children’s ailments. Not having a system in place to make interpretation services available at all healthcare providers denies families the vital communication link that they need to maintain their children’s health.

Immigrants and refugees come from a variety of countries with varying levels of development and access to health services. Some people immigrated from countries where access to healthcare was very scarce and only available to those who could pay for private services. Some participants did not know how to navigate the Canadian healthcare system. This lack of information about how healthcare works in Canada meant that they did not take advantage of available services which led to unnecessary difficulties. For example, one participant did not know that if interpretation services were available at a health facility, she would not have to pay for them:

I don’t know, and I think—okay, what I heard from online, there’s some information. They say if you, you know, you totally can’t speak English, if you go to a hospital they will give you or there are other people who can to help you interpret your situation. But I thought it’s just something maybe for—but not common, let’s say.

(Participant 025, Mom)

Another parent, who came to Canada as a refugee from Syria, had a child with mental health problems and needed assistance. She explained via an interpreter that she did not know where to turn to:

Yeah. Yeah, the age for her daughter, she needs some counselors and advices from the professional. She doesn’t want—she doesn’t know where should go or ask who’s will help her with that. So this is something she concern.

(Participant 019, Mom)

Even parents who had been in Canada for a long time sometimes did not know where to turn to for medical assistance. One participant who had lived in Canada for 15 years was still unsure of how to navigate the healthcare system. Additionally, participants mentioned not knowing when it was best to consult a family doctor or go to a walk-in clinic rather than the emergency room for certain illnesses. Thus, inadvertently they were contributing to unnecessary traffic in the emergency rooms. Other participants did not know that their extended insurance covered certain medical expenses and so paid out of pocket; they found the process of trying to claim for the expenses complicated and daunting.

### 3.4. Relationship with Health Professionals

Participants want health professionals to spend more time listening to them and trying not just to understand the specific complaint they were visiting for, but also to get to know them as individuals. This suggests an unmet desire for a relationship where they feel the health professional is treating not just an ailment but also a person, and where the health professional cares about them as a human being. This emphasis on health professional–patient relationship building is critical because it can help health professionals understand underlying root causes of patients’ illnesses or symptoms. One participant explained the dissatisfaction with the patient–doctor relationship thus:

I appreciate it when doctors try to get to know their patients a little bit, because I believe that there are things outside, like there are other factors, other aspects of people’s lives that affects their health, just instead of just looking at the symptoms of maybe what a person is suffering from. So I would have liked a family doctor who would show a little more interest in–not to pry, probe in my personal life too much, but at least show a little concern and interest in what goes on, to be able to get maybe—that would even give you more ideas as to how maybe something is coming up, but.

(Participant 050, Mom)

Many participants come from communal cultures with a strong emphasis on relationship building and maintenance where health professionals took the time to get to know them; they feel disappointed when that emphasis is absent in Canada. Many felt that doctors in Canada were always in a rush and did not spend enough time with patients to get a holistic understanding of their health and ailments, which led them to treat symptoms in isolation without getting to the root of the problem. This search for relationship is also exemplified in Participant 002′s experience where his daughters wanted to show their appreciation for their dentist by giving him a Christmas card but were prevented by the receptionist from delivering it to him themselves because they did not have an appointment:

My kids—they together want to give a card for the pediatrician. And then they see their dentist, and then pediatrician on the same day. This is December 20-something. And then his—his secretary, we ask to give him a gift card. Every year they go there. She said, “Doctor is not ordinary person. You can’t see him,” she said. And then we are not happy anyways. Because I told them to see their doctor. They are exciting to give him a gift. But because of that, they didn’t see, and then they’re asking me, “Where is my doctor?” That is—it’s not a good experience.

(Participant 002, Dad)

Additionally, the reluctance of doctors to prescribe medication is a dimension that influences the relationship with healthcare professionals. Participants often lacked understanding of why doctors did not prescribe medications that they requested. Specifically, many parents had a problem with doctors not prescribing antibiotics for children when they had a cold or fever. They shared that in their countries of origin their children would be prescribed antibiotics on the first visit to the doctor if they had cold or flu symptoms and were frustrated that Canadian doctors advised them to use over-the-counter medication or to allow the symptoms to run their course. This frustration caused some parents not to take their children to the doctor but to treat them at home because they felt that even if they took them to the doctor, he or she would not prescribe anything that they could not get over the counter. Participant 006, a mother, explained,

Yeah, because every time when I go to the doctor with the infant kids, especially, they didn’t give us anything. So I just put her at home on the Tylenol, Advil, and so I start, and so it’s better, I don’t need to go there.

The problem here is that parents are not trained to assess illnesses and provide correct treatment, so there may be times when a child needs to see a physician but the parent decides to use over-the-counter medication and the child’s illness worsens.

### 3.5. Financial Barriers to Access

Immigrant parents’ ability to access healthcare differed according to class. Those who had more financial resources or were employed in jobs that gave them extended healthcare were better able to afford prescription drugs and other resources that their children needed. Immigrants and refugees are often employed in lower-wage jobs because their credentials may not be recognized, and they do not have Canadian work experience. This means they have a lower income than the average Canadian-born person, and often have little or no room for any extra spending. Many parents indicated that although some health services are free in Canada, those that are not covered are prohibitively expensive for them. Many stated that they could not afford medication, especially if they did not have extended healthcare.

Finances also impacted parents’ access to mental healthcare for their children. The cost to see a private mental health practitioner is high and the service is not covered under the Alberta Health Care Insurance Plan. Thus, lower-income parents must forego this treatment for their children. One parent, who was the mother of two children, found the high cost of accessing private counselling prohibitive, lamenting:

And then there’s really nothing for this age. You know, counsellor, to get a counsellor for a kid, oh, my God. Mostly are private. $200 a session. How can I pay 200 bucks? I cannot even pay for myself, $170, $180, whatever it is. And then not everyone works with kids. Doctors, all they can do is refer, refer. But there’s nothing. Where do they refer you to?

(Participant 049, Mom)

People have several avenues to access a doctor for free for physical ailments, but there are few affordable options to see a mental health practitioner. Although subsidized services are offered through nonprofit organizations, the waiting lists are long and can extend up to eight months. Sometimes, participants were told they are not receiving new patients, and some participants who used these services felt that the care was substandard. This lack of access to affordable mental healthcare is critical for refugees, many of whom have experienced significant trauma and suffer from a variety of mental illnesses as a result. Denying them the help that they need constrains their ability to integrate into Canadian society and achieve to their full potential.

Parents explored the option of purchasing private healthcare as a way of getting their children the necessary help but the cost of that was prohibitive also. As one participant remarked,

So I looked into like Blue Cross private coverage. You pay for—you—like the minimum that I would pay, it’s like $1500, $2000 a year, and they only give me—they only cover $500 dollars for psychology. That’s not enough. And not only that they only cover $500; they say, “We only pay $30 per each session.” So out of $200, I’ve got to pay $230 dollars—sorry, $130 dollars. I still can’t afford it.

(Participant 049, Mom)

Finances pose even more of a barrier for undocumented immigrants as they do not have provincial health insurance and must therefore pay for services out of pocket, which puts healthcare out of their reach. Without insurance, healthcare in Canada is expensive. Additionally, some undocumented immigrants may fear going to the health professional because they feel they may be deported. Thus, many live with untreated illnesses. One participant filed an asylum claim in Canada. She had five children, four of whom were born outside of Canada and did not have provincial health insurance. One of the children was asthmatic; she did not have an inhaler or any other basic asthmatic medication and did not see the health professional regularly because of the mother’s inability to pay and the long distance of the free clinic for uninsured persons from their homes. In the end, these families are unable to access the help they need for their children because they cannot pay for it:

The chance to get to them is very hard. If you drive, and if you get somebody who give you a ride all the time, maybe it’s okay. But it’s very hard. And I never take my children to hospital because of this problem. I don’t have any health card. And even if they are sick, I don’t think I can take them now.

(Participant 022, Mom)

## 4. Discussion

Overall, participants faced several systemic challenges in accessing healthcare, including long wait times, lack of widespread interpretation services, and financial difficulties. Other challenges included different cultural expectations for health professionals and lack of knowledge about how to navigate the Canadian healthcare system. These are less prevalent among the Canadian-born because, having lived in Canada for much if not all of their lives, they learn about their benefits and they are fluent in at least one official language, so communication is not an issue. Our findings are consistent with previous research on access to healthcare for immigrant populations [11,13,14]. Also consistent with other studies [16,17,18,19,21], we found that language was a dominant barrier in access to health services.

Like other studies [16,17,36], we found that the lack of interpreters at some health facilities was a barrier for some participants who had low English proficiency. Healthcare in Alberta is provided largely by Alberta Health Services. There is generally no access to in-person interpretation services as this service was discontinued by the provincial government years ago. However, immigrants and their care providers can access a remote interpretation service. Some immigrants find that lack of access to in-person interpretation is often a barrier as remote interpreters are unable to assess the nonverbal cues of parents and children. Also, some health professionals do not use interpretation services. This limited the participants’ ability to communicate their children’s health issues to healthcare professionals and their ability to schedule visits as needed, unless they received assistance from a family member or friend. Also, participants were concerned about issues of confidentiality and privacy as they were reluctant and sometimes avoided discussing sensitive issues about their children’s health or their family issues when a friend or family member interpreted for them, fearing that they might divulge the information to other people.

Another barrier was participants’ lack of familiarity with the Canadian healthcare system. The participants came from various countries with healthcare systems that are organized differently from Canada in terms of payment for health services, the types of services patients had access to, and the procedures for patient care. Likewise, other research [16,17,36] found that immigrant parents were unaware of the free benefits they were entitled to under Canada’s universal healthcare system. Inadequate knowledge of available free or subsidized services prevented some parents from accessing mental health supports and dental care for their children.

Although provincial insurance plans offer many health services free to immigrants and refugees who are covered, some parents shared that lack of finances prevented them from accessing some services that are not covered, such as some forms of psychological counselling services in outpatient settings and professional dental care. This reinforces the findings of previous research [29,30] which found that low-income immigrant families are unable to afford dental insurance for their children, which results in immigrant children not receiving professional dental care. Other studies, including [15], also identified finances as an impediment to immigrant families accessing healthcare services for their children.

There were several areas of divergence between the present study and the studies consulted in the review of literature. One area of divergence was that the participants in our study identified long wait times in between booking an appointment and seeing the health professional as well as at healthcare clinics as an impediment to seeking care. Some either treated their children with home remedies or over-the-counter drugs or delayed taking them until symptoms worsened in an attempt to avoid the long hours. This issue was not highlighted in the studies we reviewed [16,17,29,37]. Further, our study participants indicated that they desired to have stronger patient–health-professional relationships and identified doctors’ reluctance to prescribe antibiotics and other strong medications as a deterrent to them seeking care for their children. These issues were not explored in the studies we consulted.

## 5. Conclusions

Because our study used a qualitative approach, we do not seek generalization but transferability of our findings. Our findings provide several implications for policy, research, and practice. First, policymakers need to address issues related to long wait times for health services in Canada. Increasing the supply of health professionals is one solution. Policymakers and service providers should also recognize that income is a determinant of health. Addressing the underlying causes of income inequity between immigrant and nonimmigrants, such as challenges with credential assessment, will eventually contribute to access to healthcare. Also, funding should be available to hire trained interpreters to assist immigrant populations in health settings, which will ensure that immigrants receive appropriate information. Moreover, developing a therapeutic relationship with immigrants is vital. Showing respect and dedicating time to address the complex health and settlement challenges immigrants face will improve access to healthcare. Further research should consider using quantitative data to provide generalizable knowledge on access to healthcare for immigrant children. Also, further research is needed on strategies to improve access to healthcare for immigrant children. Likewise, more research is needed on strategies to fill information gaps for immigrant populations.

## Figures and Tables

**Table 1:** Demographic Description of Participants.

Participant Code	Country of Birth	Length of Time in Canada (Years)	Migration Path to Canada	Highest Level of Education	Family Income (Canadian $)
OO1	Nepal	4.6	Economic or skilled immigrant	College certificate/diploma	30,000–50,000
OO2	Ethiopia	9	Refugee, privately sponsored	College certificate/diploma	50,001–70,000
OO3	Ethiopia	3.6	Refugee, privately sponsored	University degree	50,001–70,000
OO4	Pakistan	12	Family-sponsored immigrant	High school or less	less than 30,000
OO5	Nepal	1.11	Economic or skilled immigrant	Postgraduate degree	30,000–50,000
OO6	Pakistan	3.6	Family-sponsored immigrant	College certificate/diploma	30,000–50,000
OO7	Syria	2.4	Refugee, privately sponsored	University degree	30,000–50,000
OO8	Egypt	5	Family-sponsored immigrant	College certificate/diploma	less than 30,000
OO9	Iraq	8	Refugee, government sponsored	None	less than 30,000
O10	South Korea	3.6	Economic or skilled immigrant	Postgraduate degree	70,001–100,000
O11	South Korea	3	Economic or skilled immigrant	College certificate/diploma	100,001–150,000
O12	Nepal	2.5	Economic or skilled immigrant	Postgraduate degree	30,000–50,000
O13	Saudi Arabia	2.6	Economic or skilled immigrant	University degree	less than 30,000
O14	South Korea	6	Economic or skilled immigrant	University degree	70,001–100,000
O15	Nepal	2.6	Economic or skilled immigrant	Postgraduate degree	50,001–70,000
O16	South Korea	3	Temporary foreign worker	College certificate/diploma	50,001–70,000
O17	Iraq	0.3	Refugee, privately sponsored	University degree	less than 30,000
O18	China	20	Family-sponsored immigrant	College certificate/diploma	150,001 and above
O19	Syria	0.7	Refugee, privately sponsored	College certificate/diploma	less than 30,000
O20	Ethiopia	4	Refugee, government sponsored	University degree	less than 30,000
O21	China	16	Family-sponsored immigrant	University degree	100,001–150,000
O22	Ethiopia	1.6	Came on visitor visa and applied for asylum after	High school or less	less than 30,000
O23	Ethiopia	5	Refugee, privately sponsored	College certificate/diploma	70,001–100,000
O24	South Korea	1.6	Family-sponsored immigrant	Postgraduate degree	50,001–70,000
O25	China	4	Economic or skilled immigrant	Postgraduate degree	30,000–50,000
O26	India	1.5	Temporary foreign worker	Postgraduate degree	50,001–70,000
O27	Libya	3	Refugee, government sponsored	University degree	less than 30,000
O28	Iraq	3.6	Refugee, government sponsored	College certificate/diploma	less than 30,000
O29	China	7	International student	Postgraduate degree	50,001–70,000
O30	India	7	Family-sponsored immigrant	Postgraduate degree	70,001–100,00
O31	Philippines	24	Family-sponsored immigrant	College certificate/diploma	100,001–150,000
O32	Nigeria	6	Economic or skilled immigrant	Postgraduate degree	100,001–150,00
O33	China	2.6	Temporary foreign worker	Postgraduate degree	30,000–50,000
O34	China	0.6	International student	Postgraduate degree	less than 30,000
O35	Ghana	2	International student	Postgraduate degree	30,000–50,000
O36	Philippines	12	Family-sponsored immigrant	University degree	100,001–150,000
O37	China	14	Temporary foreign worker	High school or less	less than 30,000
O38	China	10	Family-sponsored immigrant	College certificate/diploma	70,001–100,000
O39	Vietnam	4	Family-sponsored immigrant	Other: primary grades	30,000–50,000
O40	India	0.9	Family-sponsored immigrant	Postgraduate degree	less than 30,000
O41	Iraq	4	Economic or skilled immigrant	University degree	50,001–70,000
O42	Iraq	chose not to answer	Refugee, government sponsored	High school or less	Chose not to answer
O43	Ukraine	17	Family-sponsored immigrant	University degree	70,001–100,000
O44	India	0.3	Economic or skilled immigrant	College certificate or diploma	less than 30,000
O45	Nigeria	2	International student	Postgraduate degree	30,000–50,000
O46	India	1	Economic or Skilled Immigrant	University degree	less than 30,000
O47	Liberia	1	Economic or skilled immigrant	University degree	less than 30,000
O48	Nigeria	3	International student	University degree	30,000–50,000
O49	Romania	15	International student	University degree	less than 30,000
O50	Ghana	5	Temporary foreign worker	Postgraduate degree	50,001–70,000
